# Adherence to Self‐Management for Chronic Diseases Among Unemployed Jobseekers in Finland—A Qualitative Study

**DOI:** 10.1111/scs.70230

**Published:** 2026-04-06

**Authors:** Nina Kemilä, Outi Kähkönen, Leila Paukkonen, Anne Oikarinen

**Affiliations:** ^1^ Research Unit of Health Sciences and Technology, Faculty of Medicine University of Oulu Oulu Finland; ^2^ Department of Nursing Science University of Eastern Finland Kuopio Finland

**Keywords:** adherence, chronic diseases, jobseeker, self‐management, unemployment

## Abstract

**Aim:**

The aim of this study was to identify the factors that promote and weaken adherence to self‐management among unemployed jobseekers with chronic diseases.

**Design:**

A qualitative descriptive study was used.

**Methods:**

The study participants (*n* = 12) were recruited from users of employment services, and data were collected during summer 2022 via semi‐structured thematic interviews and analysed using inductive content analysis.

**Results:**

Three main categories emerged that promote adherence to self‐management of chronic disease: functioning of the service chain, psychosocial support and life management. Furthermore, two main categories were identified as weakening adherence to self‐management of chronic disease, namely, challenges in coping with everyday life and challenges related to access to services and information.

**Conclusion:**

Adherence to self‐management is influenced by both individual and disease‐specific factors, as well as the available resources. Unemployment does not undermine adherence to self‐management, yet the harmful aspects of unemployment can strongly affect an individual's ability to cope and take care of themselves. Unemployed people with chronic diseases want to participate in the decision‐making related to self‐management but need support from social and health care services. As such, adequate health care services, professional support and coordinated service guidance will improve adherence to self‐management among the unemployed.

## 1 Introduction

Unemployment has an adverse impact on individuals' health, increasing stress, reducing mood, and potentially leading to unhealthy lifestyle behaviours [1]. It is a major socio‐economic determinant of poor health and well‐being, associated with reduced living standards, loss of social status, heightened economic and social insecurity and widening health inequalities [2]. Unemployment affects both mental and physical health in broad and interconnected ways [2, 3]. It increases the risk of developing chronic diseases, which may, in turn, exacerbate difficulties in gaining or maintaining employment, creating a reinforcing cycle of disadvantage. Research further suggests that individuals living with chronic mental illness face an elevated risk of unemployment compared to the general population [4].

According to the International Labour Organisation (ILO) [5], unemployment is defined as a situation in which an individual has actively sought work for at least 4 weeks and is available to begin employment within 2 weeks. This definition also covers individuals who have been laid off and are actively seeking new employment within 3 months. Moreover, evidence links multimorbidity (i.e., the coexistence of two or more chronic conditions) to reduced functional ability, resulting in individuals with chronic diseases often being excluded from the labour market [6]. Previous findings suggest that unemployment and multimorbidity are associated with greater susceptibility to suboptimal self‐management [7].

Health disparities further shape socioeconomic status and everyday living conditions, creating substantial barriers for disadvantaged individuals seeking to adhere to self‐management practices and adopt healthier behaviours [5, 8, 9]. Access to health services, both their availability and practical reach, plays a critical role in reducing these inequalities and mitigating the adverse consequences of unemployment [8].

This study adopts a person‐centred and holistic perspective, recognising unemployed jobseekers with long‐term health conditions as individuals with distinct experiences, needs and inherent worth. It underscores the value of respectful and compassionate encounters, as well as care models that integrate the physical, emotional, social and spiritual dimensions of well‐being while attending to each person's lived and subjective reality [10, 11]. Drawing on the Patient‐Centred Nursing Framework, patient‐centredness is understood as the capacity to engage with individuals' motivation, sense of responsibility, access to information and support networks, while acknowledging how contextual and attitudinal factors shape care processes. Within this framework, individuals are positioned at the centre of self‐management practices, offering an essential lens for examining adherence among vulnerable populations such as long‐term unemployed persons living with chronic illness [12].

Early support in healthcare and prevention efforts also helps reduce societal costs [13], as individuals with higher morbidity rates than those in the labour force underscore their greater need for services [6]. In Europe alone, an estimated 50 million people have multimorbidity [14]. This health issue, which includes conditions such as cardiovascular diseases, diabetes and mental health problems, places a substantial burden on individuals, families and society [15]. In addition to the challenges faced by healthcare services, it is crucial to understand the experiences of unemployed individuals to adequately engage with and provide care for them, particularly in supporting their self‐management of their disease(s).

In this study, adherence to self‐management of chronic diseases is defined according to Kyngäs' theory [16] as an active, intentional, and responsible process of care, in which the patient works to maintain their health in close collaboration with healthcare professionals, guided by professional counselling and tailored to the individual's situation [16, 17, 18, 19]. This definition underscores the patient's expertise, experience in self‐management, autonomy, problem‐solving ability and participation in decision‐making [16]. While self‐management refers to the day‐to‐day management of chronic diseases by individuals, professional support is a central component of self‐management [20, 21].

Further, the concept of adherence to self‐management has evolved, replacing the older compliance paradigm with an active, patient‐centred approach that emphasises the patient's agency, responsibility and collaboration in care [16, 19, 22]. While compliance reflects the extent to which a patient follows orders from professionals, adherence emphasises collaboration and a shared understanding between professionals and the patient and thus focuses on active patient participation [23], and underscores the patient's expertise, autonomy, and problem‐solving ability [16]. Another related, evolving concept is concordance, which, however, is more focused on the relationship between patients and professionals, while patient adherence is more of a behavioural process [19, 23]. The individual is responsible for their health, but the management of illness‐related matters requires support from healthcare personnel and society [24]. Healthcare professionals play a role in supporting patients' adaptation and treatment [25, 26]. Patient‐centric factors that promote adherence include motivation, responsibility for care, access to information, and support from family, friends and healthcare personnel [7, 27]. Energy, willpower, perceived results, sense of normality and fear of complications also influence adherence [15, 16, 17].

Additionally, the availability of health services [8] and multi‐professional cooperation [28] are relevant, with information, knowledge, motivation and encouragement from healthcare professionals playing crucial roles [29]. It has been shown that self‐management guidelines, as well as combining self‐management programmes with regular care and counselling, improve health‐promoting symptoms and behaviours among patients, symptom treatment, and coping with unemployment [14]. Nevertheless, there is a need for research from the perspective of unemployed jobseekers with chronic illness—an underrepresented group in existing research. The aim of this study was to identify the factors that promote and weaken adherence to self‐management among unemployed jobseekers with chronic diseases.

## 2 Materials and Methods

### 2.1 Design

The research applied a descriptive qualitative design [30]. The Consolidated Criteria for Reporting Qualitative Research (COREQ) checklist was used to guide the research and reporting process [31]. The adoption of a descriptive, qualitative approach, incorporating interviews, enables a deeper understanding of a phenomenon that has received limited research attention: the experiences of unemployed jobseekers with chronic conditions regarding their adherence to self‐management.

### 2.2 Setting and Participants

The inclusion criteria were an unemployed jobseeker living in northern Finland and a diagnosis of a chronic disease(s) such as depression, diabetes, hypertension, epilepsy, asthma or musculoskeletal disease. Interviewees were recruited by social workers from clients of the employment service in a small city in northern Finland. In Finland, unemployed jobseekers are offered free employment services and health check‐ups in outpatient care as part of the publicly funded social welfare and healthcare system [22].

A total of four men and eight women participated in this study. The study participants were aged 35–60 years. The length of unemployment ranged from 4 years to periods extending across nearly the individual's entire adult life. Some individuals had no vocational training or had never held a job or been in a permanent employment relationship corresponding to their education. They all had at least two chronic conditions, indicating multimorbidity. The most common diagnoses were musculoskeletal disorders, diabetes and hypertension (Table 1).

**TABLE 1:** Interview themes and the questions posed to interviewees.

Theme	Questions
Background information	gender, marital status, duration of unemployment, working career and education, diagnosis(s) What experiences do you have of treating your illness? How do you feel about your health? What are your health goals? What thoughts or feelings arise when you get sick? How would you describe your life after being diagnosed?
Meaning of illness	Describe your own illness, how do you experience it? What challenges or problems have you faced because of your illness? What experiences are involved in treating your illness? How do you feel about your health? What are your health goals? How would you describe your adherence to treatment? What thoughts or feelings arise when you get sick?
Meaning of adherence to self‐management	How do you treat yourself? In which types of situations is compliance with the treatment guidelines easiest? In which types of situations is compliance with the treatment guidelines the most difficult? Please evaluate your adherence.
Factors that promote/support adherence to self‐management	What skills should health professionals have to support your own care? What kind of support would you need to promote adherence to self‐management? What kind of support have you received from health care services?
Factors that weaken adherence to self‐management	What are some of the things that prevent your illness from being treated? Please clarify the factors that weaken adherence to self‐management.
How do your loved ones react to your illness?	

### 2.3 Data Collection

The interviews (*n* = 12) were conducted between June and September 2022. Convenience sampling was used [32]. Potential study participants received a cover letter and, if they were interested in participating, were asked to provide a telephone number at which the researcher could contact them. The leading researcher conducted the individual semi‐structured interviews in the employment services nurse's office. The interview themes were based on a theoretical framework (Table 1). First, the researcher introduced herself and explained the study's purpose: to build trust. The participants received oral and written information about the study, including its voluntary nature, from the first author. Before being interviewed, the participants provided written informed consent. Recruitment, interviewing and analysis were conducted concurrently, and data collection ceased once saturation was reached (i.e., the material no longer yielded new insights). The interviewer's prior familiarity with the topic facilitated recognition of data saturation.

The interview questions followed an interviewee‐oriented progression, aiming to foster a relaxed, open discussion that lasted 22–45 min. The interviews were characterised by considerable depth and multidimensionality. Their extended duration, openness, and reflective tone enabled a comprehensive and nuanced exploration of the research question, grounded in the participants' lived experiences. As a result, the generated data were of high quality, offering detailed insights into the phenomenon. After the interviews, the recordings were transcribed verbatim and participant identifiers were encoded to ensure anonymity [31]. The interview transcripts totalled 81 pages (formatted in Times New Roman, 12‐point font, single‐spaced). An example of the analytical process applied to the interview transcripts is presented in Table 2.

**TABLE 2:** Example of the analytical process applied to the interview transcripts.

Quote	Initial code(s)	Category	Main category
‘At the doctor's appointment, he [the doctor] wrote a medical statement, and I thought that we would discuss it together, and yes, he listened and then wrote it all’. (W3)	Felt the doctor did not listen	Being heard	Psychosocial support
‘They will make sure that I have taken the medicines that I have eaten and follow how I have slept’. (M4)	The social network supports daily functioning	Social network	
‘Nurses have good knowledge and are able to influence quite a few things’. (M4)	Nurses' skills and knowledge	Professional expertise	Functioning of the service chain

### 2.4 Data Analysis

The data were analysed using inductive content analysis [30]. The transcribed material was read through several times to gain an understanding of the overall phenomenon. After an initial examination of the material, a sentence or set of sentences was selected as the unit of analysis to ensure that the described experiences would reflect the study context. The selection and interpretation of data were guided by the research questions to ensure relevance. The first author analysed the data, and the co‐authors confirmed all the findings. The original statements (*n* = 207) were extracted from the transcribed material, then reduced and encoded numerically, facilitating a return to the original expressions as the analysis progressed. Following the reduction, these expressions were condensed to capture their essential meaning and then grouped into subcategories with similar content. In the next step, the subcategories (*n* = 37) were categorised into upper categories (*n* = 17) based on similarities in content. Finally, the upper categories were further organised into main categories (*n* = 5). Appropriate names were assigned to these categories to represent their thematic significance. An illustration of the data analysis is given in Table 3.

**TABLE 3:** Sociodemographic data of the participants (*n* = 12).

Variable	% (*n*)	Mean	SD
Age		48	25
Gender			
Female	66.6 (8)		
Male	33.3 (4)		
Marital status			
Single, divorced	58.3 (7)		
Married, cohabit	41.6 (5)		
Diagnosis			
Musculoskeletal disorder	66.6 (8)		
Diabetes (type 1 or 2)	50.0 (6)		
Hypertension	41.6 (5)		
Depression	33.3 (4)		
Eczema	16.0 (2)		
Epilepsy	16.0 (2)		
Morbid obesity	16.6 (2)		
Coronary artery disease	16.6 (2)		
Number of chronic conditions			
One	0.0 (0)		
Two or more (multimorbidity)	100.0 (12)		
Unemployment status			
Unemployed jobseeker	41.6 (5)		
Unemployment, sick leave	25.0 (3)		
Unemployment, rehabilitation work	16.6 (2)		
Unemployment, work trial	8.3 (1)		
Unemployment, part‐time	8.3 (1)		
Duration of unemployment			
Under 12 months	8.3 (1)		
12–36 months	16.6 (2)		
Over 36 months	41.6 (5)		
Never been in permanent employment	25.0 (3)		
Profession			
Customer service work (commerce/restaurant)	41.6 (5)		
Entrepreneur	16.6 (2)		
No occupation/vocational training	16.6 (2)		
Industrial work	8.3 (1)		
Care profession	8.3 (1)		
Other	8.3 (1)		
More than one profession	41.6 (5)		
Education			
Comprehensive school	100.0 (12)		
Vocational school	50.0 (6)		
Trade school	16.6 (2)		
High school	8.3 (1)		
No education after comprehensive school	25 (3)		
More than one form of education	16.6 (2)		

### 2.5 Ethical Considerations

The study was conducted in accordance with good scientific practice [33]. The study organisation granted research permission. According to Finnish legislation, the study did not require approval from a Research Ethics Committee because it does not involve minors and will not cause direct or indirect physical or psychological harm to the participants [34]. The study also complied with the European Union's General Data Protection Regulation [35]. Each participant received an invitation letter outlining the study's purpose, the voluntary nature of participation, confidentiality, anonymity and data handling. Written consent was obtained from all participants. All participants' personal information was kept confidential, and anonymity was ensured when reporting the findings. Participants were informed of their right to withdraw from the study at any point without any consequences. The transcribed research data will be retained until publication of the research results.

The participants in this study comprised a particularly vulnerable population, a factor that was carefully considered throughout the study's design and implementation. Some participants were previously known to the researcher through prior therapeutic relationships. While this familiarity fostered trust and openness, it also necessitated heightened ethical vigilance and reflexivity. To mitigate potential bias and maintain professional integrity, the researcher relied on clinical expertise, situational awareness and ongoing collegial consultation with the research team. Interviews were conducted face‐to‐face in settings familiar to and perceived as safe by the participants, thereby facilitating comfort and supporting transparency during data collection. The face‐to‐face format further enabled the researcher to attend to and reflect on participants' emotional expressions, thereby enriching the depth and quality of the data.

## 3 Results

The analysis of the collected data identified three main categories related to factors that support self‐management: psychosocial support, functioning of the service chain and life management; and two main categories related to factors that weaken adherence to self‐management: challenges in coping with everyday life and challenges of access to services and information. The results are presented in Tables 4 and 5.

**TABLE 4:** Factors that promote adherence to self‐management for chronic disease.

Main category	Category
Psychosocial support	Being heard Social network Support for activities and inclusion
Functionality of the service chain	Professional expertise Continuity of care Functionality of the service system
Life management	Acceptance of their situation The importance of meaningful things for well‐being Adherence to a healthy lifestyle

**TABLE 5:** Factors that weaken adherence to self‐management for chronic disease.

Main category	Category
Challenges in coping with everyday life	Lack of support from loved ones Loss of functional capacity Experiences of ineffective treatment Economic barriers Problems with medication
Challenges of access to services and information	Lack of interaction in the patient relationship Lack of access to information
	Challenges arising from the service system

### 3.1 Factors That Promote Adherence to Self‐Management for Chronic Disease

#### 3.1.1 Psychosocial Support

Psychosocial support experiences encompass being heard, having a social network, and receiving support for activities and inclusion. Being heard in psychosocial support involves influencing self‐management decisions and promoting positive interactions. Examples included doctors listening to opinions, discussing treatment plans collaboratively and tailoring them to individual needs. Positive encounters were characterised by understanding, friendly treatment and supportive attitudes from professionals and nursing staff.

At the doctor's appointment, he [the doctor] wrote a medical statement, and I thought that we would discuss it together, and yes, he listened and then wrote it all. (W3)

A social network comprises support from friends and family, including parents, spouses, children and friends, all playing meaningful roles in coping with chronic disease. Family and friends are understanding and accustomed to their loved ones' illness, eliminating any feelings of shame regarding treatment measures in their presence. Children and grandchildren serve as motivators for maintaining health and functionality. Participants emphasised the importance of being able to help their loved ones. Additionally, close relatives or friends often assist with basic aspects of daily life, such as sleeping, eating and taking medication. Peer support was also regarded as significant.

They will make sure that I have taken the medicines, that I have eaten, and that I follow how I have slept. (M4)

Support for activity and inclusion included benefits from rehabilitation and mental health services. Rehabilitation included rehabilitative work activities and illness‐specific rehabilitation arranged by the Social Insurance Institution. Participants who had been in rehabilitation mentioned having received peer support, new information and methods to promote physical and mental health. Support for mental health services involved various types of therapy, supportive discussions and various treatment experiments at the psychiatric clinic.

First, it's nice yeah, and then I ironed and did laundry there, and if you started to feel like you couldn't do it anymore, you could switch to another job. It was wonderful. (W3)

#### 3.1.2 Functioning of the Service Chain

The functioning of the service chain included professional expertise, continuity of care, and the functionality of the service system. The participants described professional expertise in terms of the quality of the information they received from health and social services professionals. According to the experiences of unemployed participants, social welfare professionals provided the most accurate information about their situation. The information provided by public health nurses, physiotherapists, and pharmacy staff was described as understandable and reliable.

Nurses have good knowledge and are able to influence quite a few things. (M4)

Access to assistance from social services professionals included information and concrete support for business activities, appointments and filling out various forms.

I didn't get any help until I got here, so the public health nurse has been the best [type of support] and the only one. (W5)

Based on the participants, continuity of care involved cooperation between professionals, receiving clear instructions and regular follow‐up. Co‐operation between professionals was described as collaboration between the nurse and the doctor; for example, the nurse had prepared the doctor's appointment in advance by consulting the doctor on questions concerning the client. Consultations between doctors were considered useful as the right type of treatment could not otherwise be found.

Last time I went to the doctor, it was the same young boy as had consulted me before. But he called the dermatologist and then ordered those pills [that helped my condition]. I love that he called another doctor when he saw that the previous treatment was not working. (W3).

According to the experiences of the participants, getting clear instructions was beneficial to choosing the right treatment and getting better self‐management results. Moreover, the participants felt that receiving tasks made it easier for them to focus on self‐management when decision‐making seemed difficult.

In fact, I think I'll do better when told what to do and at what time; it'll feel better for me. (M3)

Regular follow‐ups meant periodic visits to the nurse's office. This long‐term relationship sometimes lasted for several years and often even compensated for the lack of support from family members. A high‐quality treatment relationship can be sustained for a long time and supports the recovery process.

Of course, there were regular meetings with the diabetes nurse, and it was wonderful to work with her. There's still the same nurse, and I didn't have any parents I could have talked to it about. So maybe it [the care relationship] should last longer for adequate support. (W1)

The service system's functionality covered aspects such as free health services for individuals receiving labour market support for more than 500 days, access to treatment, and a seamless flow of information. Participants noted that, since unemployed individuals with basic income support received a financial commitment from the government to cover the cost of medications, this removed the economic burden of purchasing required medicinal products. The availability of treatment refers to an effective system of appointments, the availability of reception times, and adequate health services. The final aspect of functionality—seamless information flow—involved transferring patient data to the patient information system, enabling the doctor to assess any relevant data before an appointment. The participants detailed how having a personal doctor made visits easier, as they did not have to explain their condition from the beginning during each visit.

I'm on basic income support, so I don't have to pay for those drugs ‐ if I had to go to a doctor as much as I've had to, I'd have gone bankrupt. When this case came up with my own doctor, I didn't have to tell every point of my condition to the doctor separately… He already knew what was wrong with me and all of the tests that I needed. (M2)

#### 3.1.3 Life Management

The participants described life management as the acceptance of their situation, the importance of meaningful things for well‐being, and adherence to a healthy lifestyle. The participants felt that accepting the situation reflected sufficient understanding about the condition. Any worsening of symptoms made participants realise that they would have to undergo self‐management, or risk further complications and an exacerbation of their conditions. Some of the participants described acceptance of the disease as a specific type of optimisation, or an obligation to adapt to the situation.

And then one day, when you stop taking your medicine, you'll find that your face is on fire, the sugars are really high, and your eyes are swollen. (M3)

The participants described the importance of doing meaningful things for well‐being by listing activities that were pleasurable, such as reading, outdoor activities, spending time with pets, meeting friends, travelling and taking personal time. Adherence to a healthy lifestyle included weight management, regular exercise and movement, as well as maintaining the programme. The participants described how mobility and physical training were parts of rehabilitation from surgery that could weaken joint stiffness. Weight loss was found to be more effective and successful when it was associated with strong feelings of success and increased well‐being.

Maybe I'll feel better, at least if I lose weight, I think I can do better… such a winning mood when you manage to achieve someone's goal that you've never wanted or been able to do before. (M4)

According to the participants' experiences, a regular and steady life made it easier to maintain the rhythm of everyday life, have meals at set times, and enjoy high‐quality sleep. For diabetic patients, this meant that they did not experience large swings in sugar metabolism, and anxiety levels also fell when the quality of sleep improved.

Your body is much fresher, and your mind is much fresher when you keep your sugars in check. I feel like I've been reborn. It has been such a calm life situation that I feel as if I can even eat a chocolate bar and it will not be a problem. That's been cool. (M3)

### 3.2 Factors That Weaken Adherence to Self‐Management for Chronic Disease

#### 3.2.1 Challenges in Coping With Everyday Life

Challenges in coping with everyday life were described in terms of a lack of support from loved ones, the loss of functional capacity, experiences of ineffective treatment, economic barriers and problems with medication. In certain cases, the lack of support from loved ones manifested as challenging relationships, with resources spent on resolving relationship problems.

I've had a relationship that has dominated my whole life. Until last year… a person who has, in a way, taken all of the resources. (W1)

Some of the participants felt that their family members were not interested in their illness or were not used to discussing the condition. Furthermore, some interviewees described how their disease and unemployment had caused friendships to end.

I don't know how they think I'm home. We've never talked. They know I'm on sick leave. (W4)

It is possible that some family members did not understand the severity of their loved one's illness. For instance, one participant stated that they could manage their everyday affairs yet were mentally and physically in poor condition. As such, the individual did not want to ask for help and show their weakness because they were used to supporting other family members. In many cases, participants pointed out that they have to care for both their children and their parents.

I would have needed family support. I think I may have been too hard [on myself] at the time. (W7)

The loss of ability to function was described as a consequence of pain, insomnia and/or fatigue. The participants shared how pain affected both their endurance and sleep. As a result of pain, the participants may have slept poorly and then experienced even worse fatigue the next day. Pain can also weaken an individual's ability to move or perform everyday chores. As such, the participants shared that a lack of endurance negatively affected adherence to self‐management.

It's just that you can't do anything when you can't sleep. (W6).

The participants stated that fatigue has a detrimental effect on their ability to function. According to the interviewees, fatigue caused a lack of composure, decreased motivation and a low mood. Furthermore, they provided examples of how it was easier to eat unhealthy foods, forget to monitor blood sugar levels, lack the energy to complete necessary applications for subsidies, and fail to make appointments for social welfare and health services when feeling tired. The interviewees also described how fatigue caused feelings of guilt, laziness, and a sense of infallibility.

I know I should work hard on weight loss, that's what it's all about… sometimes laziness. For example, working so hard that I haven't been able to go to the gym year. I'm too tired to go to the gym after work. (M3)

Participants described ineffective treatment experiences as a lack of positive results and the development of additional diseases. Participants felt that treatment methods, such as medication, surgery, gymnastics, or self‐management, did not bring visible benefits or improve their condition. Despite treatment, some continued to experience fatigue, blood sugar fluctuations, blood pressure issues, or poor mood, indicating that the desired outcomes were not achieved. Some reported failed surgeries. The emergence of additional diseases was particularly frustrating, often disrupting employment plans. For example, one participant noted that, as they began to manage daily life, new problems arose.

Nerve root block, that's… let me be honest, it failed, and it didn't do any good either. (W8)

Economic barriers were reported as a challenge for self‐adherence to chronic disease care. Unemployment and illness had severely impacted some participants' finances, significantly affecting their healthcare. A healthy diet was often too expensive, and by the end of the month, money was scarce or nonexistent, which affected food quality and variety. Participants on unemployment support received government‐provided medications, but part‐time workers often exceeded the income limit for support, leading some to forgo prescribed medications.

Or there was that financial obstacle that, in a way, I could not eat as much, or as varied in terms of ingredients, as I should … When I've been unemployed it's the money issue … A healthy diet is very expensive to maintain, it's sometimes challenging. (M3)

Medication issues included poor access to drugs and drug‐induced adverse reactions. Availability problems arose with some antidiabetic and weight‐loss medications, leaving participants waiting several weeks for information on their provision or potential treatment interruption. Diverse experiences of adverse reactions were reported, including fears of side effects and addiction. Some participants became ill after starting new medications and discontinued therapy altogether. Fear of side effects also deterred some participants from starting psychiatric medications.

I'm afraid I'm gonna be some opioid junkie so I'll always have long lines I won't take them. (W6)

#### 3.2.2 Challenges to Access to Services and Information

The challenges associated with limited access to services and information ranged from challenges arising within the service system itself to a lack of interaction with patients and a lack of access to information. The challenges arising from the service system included issues with patient data transfer, uncertainty regarding the continuity of care, and difficulties accessing care. The participants felt that patient data were not transferred from one service provider to another or that the information was transferred slowly. Moreover, the information may have contained certain errors which caused misunderstandings about the client's situation.

Those files also have factual errors, and then there are misunderstandings in which you must always explain the problem. But they don't them. (W6)

Uncertainty associated with the continuity of treatment was often expressed through the treatment relationship with various professionals. The participants found it frustrating when they had to explain their medical condition from the beginning at each appointment. There were differences between the practices of different organisations and service providers, and sometimes this caused problems with even the most basic of issues.

This is a ridiculously bad system. I go to occupational health care; I get my meds… and then I'm out of work… I need the same… You can't renew the recipe because it's private. (W5)

Problems with access to treatment were characterised by a lack of progress, delays in obtaining appropriate tests, and a waiting period of several years. The interviewees felt that the health centre did not provide acute help because of their chronic disease diagnosis. In some cases, the symptoms were assessed as less severe than the actual situation, and as such, were not further investigated. As a consequence, the participants felt that they were alone with their illness and lost in the healthcare services.’ Because each patient had a limited number of follow‐up visits and rehabilitation appointments, many of the interviewees reported abandoning the treatment relationship.

The customer relationship was transferred away from the neurology clinic. So now it's been four years, so nothing has happened in terms of an appointment time there; I haven't even made contact myself. (W6)

According to the participants, the lack of interaction with healthcare professionals posed challenges, including being unheard, receiving inappropriate or degrading treatment, and a lack of trust in healthcare services. The participants felt that they had not been heard when describing their situation, pain or symptoms. Furthermore, the participants described how the discussions during appointments had remained superficial, and the patient's health had not been addressed in depth. For these reasons, there were situations characterised by a lack of diagnosis and/or treatment, which could potentially worsen disease progression.

Nothing really happened at that pain clinic. It was just the following drill: sit on a gym ball and take some Burana [ibuprofen]. (M1)

Inappropriate or degrading treatment was described as harsh or unkind treatment at the reception, along with negative attitudes among the medical staff. The participants felt that the medical staff rarely considered the overall situation and did not show encouraging behaviour.

When you have the same trouble for long enough you will go to a doctor. I was told by one doctor that I should consider going to see a psychologist. (W4)

The participants expressed a lack of trust in healthcare services. On the other hand, they also stated that there is no alternative but to trust a professional because they have insufficient knowledge about medical matters. When the patient‐doctor relationship was perceived as ineffective, the patient would end it. In addition, the interviewees did not trust the basic health care services.

I should contact the health centre immediately. But I don't trust the nurse responsible for epileptic episodes, so I don't think I'm going to contact them. (W6)

The reported lack of access to information encompassed challenges in obtaining information, fear and uncertainty stemming from limited knowledge, insufficient understanding of the instructions they received, and insufficient guidance. The participants described how they received information from various sources about their disease and treatment options; for example, the reception area had brochures available. However, some patients felt that the information was not comprehensive enough, while others questioned the reliability of the information.

There is little information about sleep apnea. I've only seen a doctor once, a long time ago… I've met that doctor once a long time ago. (M4)

The participants reported that the fear and uncertainty caused by the information they received prevented them from seeking further information independently. For instance, entering the symptoms into an Internet search engine sometimes provided rather shocking information. The participants also shared that reading the side effects of medicines raised concerns about the safety of the medicine.

On the other hand, if you start reading them [the side effects] very carefully, then you think that—for goodness' sake—this [medicine] causes this and that. (W6)

According to the participants' experiences, the instructions they received were not always easy to understand because the terms were unfamiliar or the context was difficult to grasp.

I mean, I usually understand everything, but sometimes there's these papers with official language which I don't understand at all. (M2)

Experiences of a lack of guidance included matters related to treating the illness, seeking benefits, making appointments, and remembering practical matters. The participants shared how they sometimes tried different medications or dietary changes independently, as there was no single correct solution to the situation.

The diabetes nurse gave me certain instructions to try because they said that other measures would not help. He couldn't give me any better advice. (W7)

## 4 Discussion

The findings of this study revealed that factors promoting adherence to self‐management in unemployed job seekers with chronic diseases include psychosocial support, service chain functionality and life management. The possibility of positively influencing decision‐making positively affects self‐management, and support from friends and loved ones helps manage discomfort and daily challenges [26, 29, 36, 37]. Previous research emphasises the crucial role of societal and familial support for unemployed individuals [3]. Again, previous studies on patients with varied chronic conditions and backgrounds have found that healthcare system support is essential for illness acceptance and treatment, aligning with the significance of support from healthcare professionals [15, 17, 26, 29]. Participants in this study who perceived that they had received valuable support from social and health professionals found that this had a positive impact on their perceptions of managing chronic illness. Encouraging encounters and informational assistance facilitated practical matters and grant applications, aligning with findings from previous studies among patients [7, 8, 28, 29, 37]. Participants in this study described the encounter as positive and encouraging, which had a beneficial effect on how they perceived and managed their illness. This experience encapsulates the core idea of caring encounters, which encompasses caring discussions and relationships and constitutes nurturing encounters [38].

The study reveals that jobseekers value professionals who are already familiar with their medical history, eliminating the need for detailed repetition during each consultation. Clear instructions from healthcare providers were considered crucial for effective self‐management. Consistent with other research, regular follow‐up visits and constructive feedback were identified as key factors in maintaining motivation, while receiving feedback encourages patients to continue the prescribed care at home [39]. This result is consistent with the findings of a previous study, which revealed that patients consider demonstrating knowledge and skills, as well as managing personal data confidentially, to be the most essential caring behaviours exhibited by nurses when providing care [40].

The study found that having up‐to‐date patient information facilitates timely handling of matters. Positive experiences of access to care and assistance are linked to adequate health services and accessible reception times, which in turn impact self‐management adherence, as noted in prior research on health inequalities between employed and unemployed individuals in northern Sweden [8]. Participants of this study emphasised the positive impact of accepting their situation and engaging in meaningful activities on coping and well‐being. Adherence to a healthy lifestyle has been reported to enhance functional ability and coping, thereby reinforcing a sense of control. This aligns with previous findings that have highlighted the link between a sense of normality and adherence to treatment [18, 28, 32].

According to this study's results, challenges in coping with everyday life hinder adherence to self‐management among unemployed job seekers. The participants felt that being in challenging relationships or the indifference of loved ones towards their illness used up their resources. Other studies have also identified the lack of social support as a debilitating factor for adherence to treatment among patients with chronic conditions [18, 41].

Study participants described how their pain negatively affected the quality of their sleep, increased fatigue and decreased coping. The fears and concerns associated with the prescribed medication also negatively affected adherence to the treatment. As previously described by Al‐Noumani et al. [42], adverse drug reactions, as well as concerns about complications, could cause patients not to take their medication. Additionally, Kähkönen et al. previously reported that treatment guidelines which are considered relevant and effective by patients will promote treatment adherence [38].

Unemployed individuals in the low‐wage sector face challenging economic circumstances. Jobseekers in the labour market report that they cannot obtain necessary medication or consult a doctor without income support. Thus, medication costs may contribute to treatment adherence problems [41]. In addition, participants reported that they may lack sufficient financial resources to maintain a healthy diet, a finding also reported in prior research [8].

The unemployed job seekers interviewed felt that challenges in accessing services and information were caused by problems within the service system and by a lack of interaction with patients. Errors in patient records could delay diagnosis or complicate treatment. Jobseekers felt uncertain about care continuity, and changes in attending professionals hindered the development of a confidential care relationship. The different operating practices of organisations also caused difficulties related to misunderstandings and uncertainty [41].

The study reveals that poor patient interaction leads to feelings of being unheard, mistreated, and mistrust in the healthcare system. Jobseekers found their appointments superficial and felt their pain or symptoms were not fully addressed. Some job seekers noted healthcare staff's lack of appreciation for prolonged symptoms and instances of arrogant or inappropriate behaviour. This is concerning, given the established role of professionals in motivating self‐management and treatment adherence. Additionally, the study identifies shortcomings in access to information, with jobseekers receiving limited and uniform information about their illness and its treatment. Some individuals had difficulty understanding the provided information, which aligns with findings by Spaling et al., who highlight the gap between instructions and day‐to‐day care [43, 44]. Previous studies have emphasised that a lack of information may jeopardise treatment adherence. The absence of counselling strongly negatively influences a patient's motivation for independent disease management, emphasising the importance of high‐quality health education for promoting well‐being and disease management [44].

This study supports earlier findings regarding the importance of psychosocial support, service accessibility, and continuity of care in promoting self‐management among individuals with chronic illness. Prior research has highlighted these factors across different patient populations. However, the current study makes a unique contribution by focusing specifically on unemployed jobseekers—a marginalised and under‐researched group whose experiences with chronic illness and healthcare systems are rarely examined in depth.

### 4.1 Trustworthiness and Methodological Considerations

According to Cuba and Lincoln (1981, 1985), the criteria that determine the trustworthiness of qualitative research are transferability, credibility, reliability, portability, validation, and authenticity [45, 46]. To increase reliability, the data collection process and the various stages of inductive content analysis were described comprehensively, allowing readers to follow the study's progression [27]. The selection and backgrounds of the participants, the research context, data collection, and the analytical process were described in as much detail as possible to increase the reliability of the research; the inclusion of authentic quotations also improved reliability [31]. The study was conducted in the Finnish context, characterised by publicly funded, integrated health and social services and structured employment support. Unemployed jobseekers are offered free health checks by public health services. Cultural values such as modesty, independence and trust in public institutions may have influenced participants' responses. While these factors limit direct transferability, the detailed contextual description supports the applicability of findings to similar welfare systems.

The study also has limitations, notably a majority of female participants and a focus on jobseekers reasonably managing daily activities. This may exclude those with significant self‐management challenges. Despite this, we achieved a diverse range of backgrounds and data saturation. The subject's sensitivity and participants' effective self‐management could introduce bias. Some might avoid discussing self‐management shortcomings due to shame, but anonymity improved research quality by providing a safe space for sharing personal experiences. Furthermore, there was a risk that the interviewer's familiarity with the situation prevented a truthful account of the current situation. However, it seemed familiarity freed up the discussion, as there was no tense researcher‐interviewer situation. Furthermore, the quality of the research material and the thoroughness of the analysis compensated for the small sample size, a generally accepted practice in qualitative research. The researcher must reflect on her role and document the various phases of the study as it progresses [32]. This motivated the use of the Consolidated Criteria for Reporting Qualitative Research (COREQ) checklist to ensure comprehensive reporting of results [31].

### 4.2 Relevance for Clinical Practice

A key novel insight revealed by this study is the significant impact of structural barriers—such as fragmented service chains, insufficient communication between professionals, and errors in patient records—on treatment adherence. These elements not only complicated access to care but also eroded trust in the healthcare system, ultimately discouraging self‐management efforts. While earlier studies have emphasised the role of social support and health education, few have addressed how systemic inefficiencies, such as frequent provider changes or miscommunication, directly influence the motivation and capacity of unemployed individuals to engage in their care. While services are in principle accessible to all, practical barriers—such as financial difficulties, bureaucracy, or discontinuity of care—can undermine commitment to self‐management.

The findings highlight the need to strengthen interprofessional collaboration across employment, health, social and third‐sector services. Professionals should be trained in multidisciplinary work and network‐based collaboration. Service coordination should be improved by appointing a clearly defined actor responsible for care pathways. Training is also necessary on service continuity and information flow, especially during transitions (e.g., from occupational to primary healthcare), to prevent care disruptions. Clients' understanding of their condition and role in self‐management must be ensured. This includes using clear, explicit, accessible materials and assessing cognitive ability to support adherence or providing guided self‐management where needed.

### 4.3 Recommendations for Further Research

Future research should adopt a multi‐method approach to deepen understanding of self‐care adherence among unemployed individuals with long‐term health conditions. Quantitative studies are needed to identify broader determinants, such as age, education and type of diagnosis. At the same time, longitudinal designs could shed light on how continuity of care and follow‐up influence health behaviours and outcomes, particularly in contexts of disrupted care. Intervention studies might test coordinated support models, such as assigning a consistent health or social care contact. In parallel, qualitative research is essential for exploring care transitions and service gaps in greater depth, particularly focusing on how social support, loneliness, and everyday functioning influence individuals' capacity to engage in self‐management. Additionally, the competence of health care professionals to support self‐management in this patient group warrants further examination.

## 5 Conclusions

This study highlights the critical importance of involving unemployed jobseekers in decisions related to their self‐management, particularly when living with chronic illnesses. Active participation, trust in professional competence and personalised guidance significantly enhance adherence to self‐management. Consistent professional follow‐up and positive healthcare experiences are essential for sustaining long‐term commitment to treatment plans. The findings emphasise the need for individualised and accessible support, as well as the development of customer‐oriented clinical pathways that ensure continuity of care, highlighting the importance of individualised and accessible support, particularly during care transitions. Strengthening interprofessional collaboration, clearly assigning care coordination responsibilities, and promoting client understanding through tailored communication are key strategies to improve outcomes. While the results align with international evidence, they also point to the need for further research into self‐management adherence among unemployed individuals.

## Author Contributions

**Nina Kemilä:** conceptualisation, methodology, investigation, formal analysis, writing – original draft. **Outi Kähkönen:** conceptualisation, methodology, supervision, writing – review and editing. **Leila Paukkonen:** conceptualisation, methodology, supervision, writing – review and editing. **Anne Oikarinen:** conceptualisation, methodology, supervision, writing – review and editing, project administration.

## Funding

The authors have nothing to report.

## Ethics Statement

Research permission was granted by the organisation in which the study was carried out. According to Finnish legislation, the study did not require approval from a Research Ethics Committee since the study does not involve minors and will not cause direct or indirect physical or physiological harm to the participants (Medical Research Act 488/1999).

## Conflicts of Interest

The authors declare no conflicts of interest.

## Data Availability

All the data were used in this study.
